# EEG-Based Mapping of Resting-State Functional Brain Networks in Patients with Parkinson’s Disease

**DOI:** 10.3390/biomimetics7040231

**Published:** 2022-12-08

**Authors:** Sarah Leviashvili, Yael Ezra, Amgad Droby, Hao Ding, Sergiu Groppa, Anat Mirelman, Muthuraman Muthuraman, Inbal Maidan

**Affiliations:** 1 Laboratory of Early Markers of Neurodegeneration (LEMON), Neurological Institute, Tel Aviv Sourasky Medical Center, Tel Aviv 6433267, Israel; 2 Department of Biomedical Engineering, The Engineering Faculty, Tel Aviv University, Tel Aviv 6997801, Israel; 3 Sagol School of Neuroscience, Tel-Aviv University, Tel Aviv 6997801, Israel; 4 Department of Neurology, Sackler School of Medicine, Tel Aviv University, Tel Aviv 6997801, Israel; 5 Movement Disorders and Neurostimulation, Biomedical Statistics and Multimodal Signal Processing Unit, Department of Neurology, University Medical Center of the Johannes Gutenberg University Mainz, 55122 Mainz, Germany

**Keywords:** resting-state networks, EEG, sLORETA, Parkinson’s disease, connectivity

## Abstract

(1) Background: Directed functional connectivity (DFC) alterations within brain networks are described using fMRI. EEG has been scarcely used. We aimed to explore changes in DFC in the sensory-motor network (SMN), ventral-attention network (VAN), dorsal-attention network (DAN), and central-executive network (CEN) using an EEG-based mapping between PD patients and healthy controls (HCs). (2) Methods: Four-minutes resting EEG was recorded from 29 PD patients and 28 HCs. Network’s hubs were defined using fMRI-based binary masks and their electrical activity was calculated using the LORETA. DFC between each network’s hub-pairs was calculated for theta, alpha and beta bands using temporal partial directed coherence (tPDC). (3) Results: tPDCs percent was lower in the CEN and DAN in PD patients compared to HCs, while no differences were observed in the SMN and VAN (group*network: F = 5.943, *p* < 0.001) in all bands (group*band: F = 0.914, *p* = 0.401). However, in the VAN, PD patients showed greater tPDCs strength compared to HCs (*p* < 0.001). (4) Conclusions: Our results demonstrated reduced connectivity in the CEN and DAN, and increased connectivity in the VAN in PD patients. These results indicate a complex pattern of DFC alteration within major brain networks, reflecting the co-occurrence of impairment and compensatory mechanisms processes taking place in PD.

## 1. Introduction

Patients with Parkinson’s disease (PD) manifest motor and cognitive deficits already in the early stages of the disease that worsen gradually throughout the disease course [1,2,3,4]. The common motor symptoms in PD include resting tremor, rigidity, bradykinesia, and postural instability [2], while cognitive impairments in PD encompass various cognitive domains including executive functions, attention, visuospatial function, and processing speed [3]. Both motor and cognitive resources are required for independent, self-sufficient everyday life, and in most functional activities these resources are intertwined [5].

By relying on the blood oxygenation level dependent (BOLD) signal, resting-state functional magnetic resonance imaging (rs-fMRI) is able to measure the spontaneous activity of the brain in organized functional and anatomical networks [6]. The spontaneous fluctuations of resting state BOLD signals are generally low frequency oscillations, observed between 0.01 and 0.08 Hz that are believed to reflect brain physiology and metabolism [7,8]. Numerous neuroimaging studies demonstrated that resting-state functional connectivity plays an important role in cognitive and motor functions [9,10]. For example, previous studies have shown that subjects with posttraumatic stress disorder (PTSD) exhibit abnormalities in resting state networks in the theta frequency band, which relates to cognitive impairments in these subjects [11]. Accumulating evidence demonstrate that with aging and disease, brain networks remodel their activity to adapt to the new situation [12,13,14]. For example, altered organization of the dorsal attention network (DAN) and unchanged ventral attention network (VAN) connectivity in PD patients with freezing of gait was associated with the effectiveness of specific interventions [13].

Seed-based approach is a common method to investigate the directed functional connectivity (DFC) between predefined regions by measuring the statistical relationship between their averaged BOLD time-course [15]. Specific alterations in DFC within sensorimotor (SMN) and cognitive networks have been described in PD patients. Changes in the SMN included decreased connectivity within the supplementary motor area (SMA) and increased connectivity in primary motor cortex (M1) in PD patients compared to healthy controls [14,16,17]. DFC changes were reported in brain networks implicated in cognitive functioning as well [18]. Nevertheless, the low temporal resolution of the fMRI-based methods and the limited frequency band that can be examined may conceal certain underlying neural processes.

The high temporal resolution of electroencephalography (EEG) and the use of a wide range of frequency bands can reveal novel insights on the construction of neural network maps related to cognitive and motor functions [19,20]. Specific changes in the power of theta, alpha, and beta bands were reported during the performance of cognitive tasks in PD patients [21,22,23]. These changes include a shift toward lower frequency bands with increased power in theta and reduced power in alpha and beta [22]. These changes have been associated with cognitive processes that are necessary for successful cognitive performance. Theta oscillations were linked to cognitive processing, working memory, and selective attention [24]. Moreover, these changes in theta power were found to be associated with generating a coordinated response in terms of readiness. On the other hand, alpha oscillations were viewed as a functional and communicative signal with multiple functions. Reduction in alpha power was attributed to higher cortical activity associated with attentional demands [25]. Beta oscillations have been suggested to reflect attentional modulation and lower beta power has been related to cognitive response [26].

Few studies investigated EEG-derived directed connectivity patterns in patients with PD, showing increased connectivity in the beta frequency band and decreased connectivity in the theta band [27]. However, these studies measured connectivity between EEG electrodes representing the sum of many underlying components generated from various brain areas (“the superposition problem”) [19,20,27]. In addition, these findings do not fully depict the functional changes within the brains’ functional networks. Recent advances in technology allow the implementation of more complex EEG analytical approaches to explore FC patterns within predefined networks.

To this end, the aim of this study was to utilize EEG-based mapping to explore DFC changes in motor and cognitive networks in PD patients in different frequency bands. We hypothesized that PD patients will demonstrate decreased DFC within the DAN and central executive network (CEN) mainly in the higher frequency bands (e.g., beta band), and increased connectivity within the VAN and SMN in low frequency bands (e.g., theta band), as compared to healthy controls (HCs).

## 2. Materials and Methods

### 2.1. Participants

Fifty-seven subjects, 28 healthy controls, and 29 PD patients, between the ages 40–90 years were included in the study. The participants were recruited from the community, the outpatient clinics of the Movement Disorders Unit and the Memory clinic at the Tel Aviv Sourasky Medical Center (TASMC). Inclusion criteria for PD patients were: (1) 40 to 90 years of age, (2) Established PD diagnosis according to the UK Brain Bank criteria, (3) Hoehn and Yahr score between 1–2, and (4) a score of ≥21 in the Mini–Mental State Examination (MMSE). Patients were excluded if they had (1) a history of previous head surgery, (2) clinical evidence or history of stroke, transient ischemic attacks, significant head injury, other unexplained or recurrent loss of consciousness, (3) neurological and neuropsychiatric disorder other than PD, (4) major depressive disorder, schizophrenia, bipolar disorder, or other psychotic disorders, and (5) epilepsy. Inclusion criteria for the HC group were: (1) 40 to 90 years of age and (2) living in the community or assisted living facility. Participants were excluded in case they reported any surgical, orthopedic, and psychiatric conditions that may prevent them from completing all aspects of the study. The study was approved by the local ethical committee and was performed according to the principles of the Declaration of Helsinki. All participants gave their informed written consent prior to participation.

### 2.2. Study Protocol

All assessments were performed while the patients were in the ON state (about one hour after last medication). All participants underwent a neurological evaluation that included the Movement Disorders Society Unified Parkinson’s Disease Rating Scale (MDS-UPDRS) and the calculation of levodopa equivalent daily dose (LEDD) to assess disease severity [28]. In addition, all participants underwent a cognitive assessment that included the Montreal Cognitive Assessment (MoCA) [29] and the Color Trail Test (CTT) [30]. EEG recording of four minutes in resting state was performed with eyes closed. We used the EGI system (GEODESIC GES 400) with 65 channels including Cz as a reference electrode at a sampling rate of 250 Hz. Electrode position was set according to the international 10-10 standard. During the EEG recordings, the participants were instructed to limit any blinking, jaw clenching, or facial expressions that may introduce artifacts into the EEG signal.

### 2.3. EEG Signal Pre-Processing

Pre-processing of the raw EEG data was performed to minimize the effects of global and local noise using the open-source toolbox analysis EEGLAB [31].

#### Global and Local Noises

Recorded EEG signal was filtered using a basic finite impulse response (FIR) filter in the range of 0.5–40 Hz. An average re-reference was used to improve signal-to-noise ratio (SNR). The primary reference electrode (Cz) and eye component electrodes were excluded from further processing. We did not detect noisy channels therefore, no other channels were excluded. The data for each subject was decomposed by independent component analysis (ICA); a method that separates the signal into additive components according to their source of brain activity. The criteria for halting the ICA decomposition was when the EEGLAB algorithm reached a level of 1E-10 of distinction between components. Noisy components derived from eye blinking, heartbeat, and muscle movements were discarded based on their activity power spectrum. This analysis was conducted using the open-source toolbox EEGLAB. For a detailed description of the algorithm see [31]. Effects of local noises that might be the result of momentary displacement of the electrode or sudden movement of the subject were scrubbed from the EEG signal using MATLAB. Signals were divided into epochs—each of 3 s (750 samples). Epochs with standard deviation (SD) higher than five were defined as noisy and were removed from further EEG analysis.

### 2.4. Source Localization Analysis

Source localization of EEG was determined using sLORETA software [32,33,34]. Brain activity for each subject was estimated based on standardized current density. sLORETA was used to create a transformation matrix based on the EGI 10-10 electrode system and to solve the inverse problem. For each subject, the localized activation volumes were extracted in each of the 750 time points (250 Hz × 3 s averaged epoch) of their recording.

### 2.5. Resting-State Functional Brain Networks Masks

**Figure 1:**
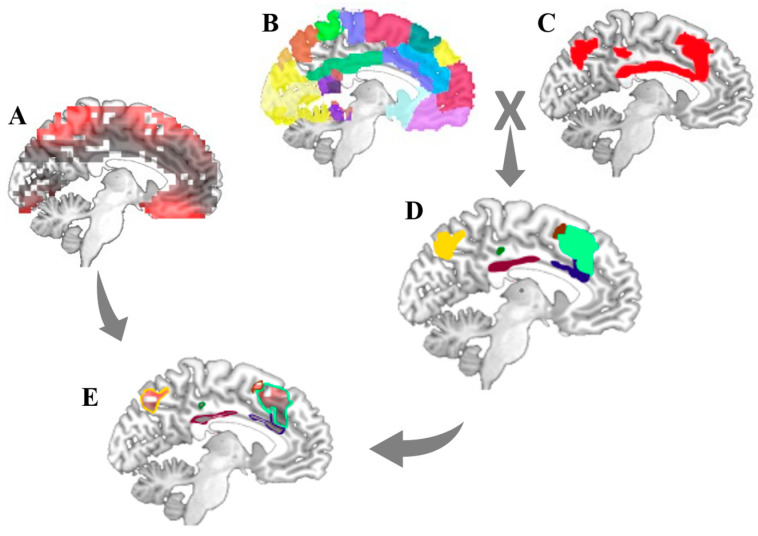
The flow chart to extract the time series of activation from each network’s hubs. (**A**) electrical activity of each voxel based on sLORETA, (**B**) MRI based binary templates of Brodmann areas maps, (**C**) MRI based binary templates of network mask (e.g., CEN), (**D**) the network’s hubs based on the intersect between network mask and Brodmann area masks, and (**E**) time series of activation in the network’s hubs.

High spatial resolution MRI-based binary templates were used to define the spatial distribution of resting state networks (RSNs) of interest (CEN, DAN, VAN, and SMN) [35,36]. A binary spatial map of each network of interest was parcellated into Brodmann areas (BAs) based on the automated anatomical labeling (AAL) atlas, producing discrete masks for the different hubs within each network (see supplementary Table S1A–D for detailed definitions of the hubs within each network). The current density of all the voxels within each network’s hubs (each voxel’s current density was calculated by sLORETA) were averaged per time-point. The implementation of these stages was performed in MATLAB using element-wise multiplication (Figure 1).

### 2.6. Temporal Partial Directed Coherence (tPDC)

Granger causality models provide causality in the time domain, allowing to assess unmeasured latent variables and exogenous inputs in the time domain in biological time-series in general, and specifically in brain signal analysis. In addition, examining the different frequency bands allows a better understanding how each band is affected in PD. To this end, temporal partial directed coherence (tPDC) method enables assessing both frequency domain as well as time domain causality [37].

The DFC measures within each network’s hubs were calculated using tPDC. This method enables the measurement of the directional information transfer between different areas of the brain in both time and frequency domains. This technique is based on dual-extended Kalman filtering and enables the calculation of a Multivariate Autoregressive (MVAR) model parameters at each time point. The MVAR model coefficients were used to calculate the causality between the time series of the brain areas and the directed coherence between them. As seen in Equation (2), using the Fourier Transform of MVAR coefficients enabled the calculation of PDC in frequency domain. The expression for an adaptive auto-regressive process can be given by the following expression:

(1) $$x\left(t\right)=\sum_{r=1}^{r=p}a_{r}\left(t\right)x\left(t-r\right)+\eta \left(t\right)$$

where $a_{r}\left(t\right)$ are the time-varying MVAR coefficients, *p* is the model order of time series *x(t)* and *η(t)* is the zero-mean Gaussian noise process.

The connectivity calculation of tPDC was as follows:

(2) $$\left|\pi_{i\leftarrow j}\left(w\right)\right|=\frac{\left|A_{ij}\left(w\right)\right|}{\sqrt{\sum_{k=1}^{k=N}{\left|A_{kj}\left(w\right)\right|}^{2}}}$$

$\pi_{i\leftarrow j}\left(w\right)$ is the amplitude of the PDC at frequency w and $A_{ij}\left(w\right)$ is the Fourier Transform of the MVAR coefficient $a_{r}\left(t\right)$. In the tPDC process, time dependent multivariate coefficients were calculated and were then used to calculate the partial directed coherence (PDC) in the frequency domain. tPDC measures were converted to time domain and were divided into theta, alpha and beta frequency bands. For additional information about the tPDC method, see [36]. To estimate the significance of a tPDC in a single network’s hub—we compared between the original tPDC and the reversed tPDC [38]. Non-significant differences between the original and reversed tPDCs indicated that the tPDC of the hub was random and not specific and therefore was not further analyzed. The algorithm for tPDC and the statistical analysis were implemented in MATLAB.

### 2.7. Statistical Analysis

The mean and standard deviation of all subjects’ clinical and demographic characteristics were calculated and evaluated for normality and homogeneity using box plots, scatter plots, and Kolmogorov-Smirnov tests. Independent *t*-test was used to examine differences between groups (PD vs. HC).

Bootstrapping is commonly used to identify random brain activity. However, due to the large number of connections in our data set, we used the time reversed method to decide if our tPDC signal was non-random [38]. We compared the coefficients from the tPDC of the brain activity time-series (“original tPDC”) with tPDC coefficients from the reversed time-series of brain activity (“reversed tPDC”). The connections that showed significant differences in the 95th percentiles after an independent t-test, followed by a False Discovery Rate (FDR) correction for multiple comparisons—Benjamin Hochberg (BH) [39], were considered for further analysis. The remaining connections were considered as random brain activity and were excluded from the analysis. For further information on this method, see [38]. For post hoc comparison results, see supplementary Table S2.

These common significant (‘real’) tPDCs were compared between the groups using independent *t*-test, followed by FDR correction for multiple comparisons. In addition, we calculated the percent of significant tPDCs within each network. Linear mixed-models were used to determine the effects of group (HC vs. PD), network (CEN, DAN, VAN, SMN), frequency band (theta, alpha, beta), and their interactions on the percent of significant tPDC while controlling for age. The number of outbound and inbound tPDCs for each network’s hub were calculated and compared between the groups using t-test and BH correction as well. The significance level for each of the described statistical analyses was *p* < 0.05.

## 3. Results

### 3.1. Clinical and Demographic Characteristics of Study Participants

Demographic and clinical characteristics of both study groups are presented in Table 1. As expected, PD patients showed significantly higher MDS-UPDRS III motor scores compared to HCs (*p* < 0.001). No differences in age, gender, and cognitive tests were observed between the study groups.

### 3.2. Within-Network Significant Connections

**Figure 2:**
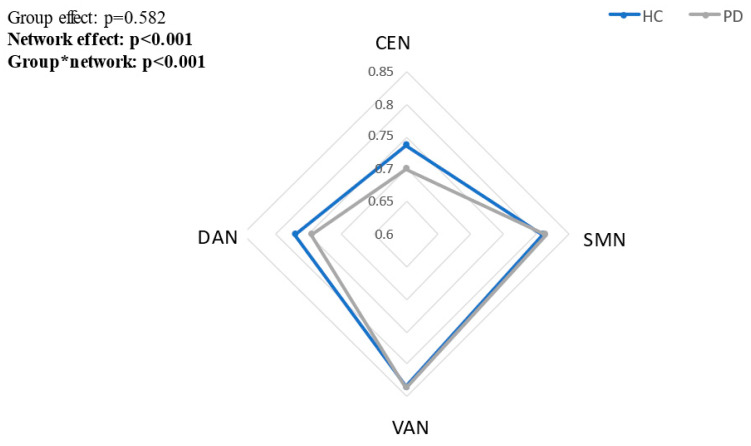
The percent number of significant tPDCs within each network in patients with PD and healthy older adults.

Patients with PD had lower percent of significant tPDCs (the amount of connections found significant using the time-reversed method) within the CEN and DAN compared to HC. No differences in the percent of significant tPDCs within the VAN and SMN were found between the groups (group × network interaction: F = 5.943, *p* < 0.001) (Figure 2). No differences in the percent of significant tPDCs were found between the frequency bands: theta, alpha and beta (group × band effect: F = 0.914, *p* = 0.401).

### 3.3. Between Group Differences in tPDCs within Each Network

**Figure 3:**
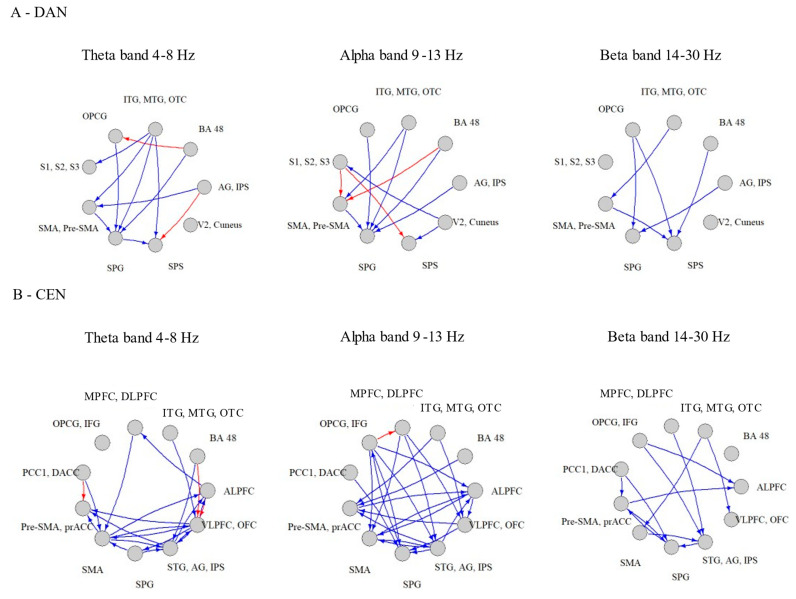

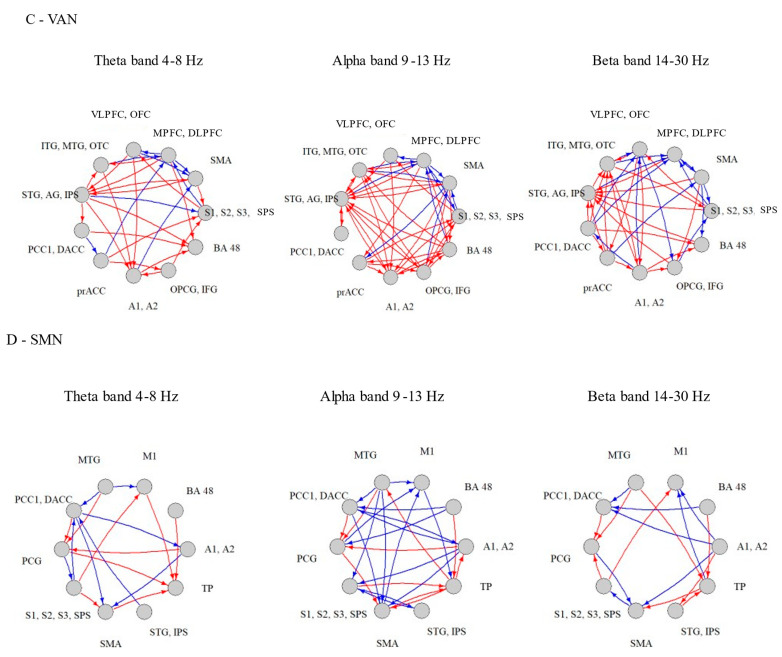
Comparison between HC and PD in the tPDC within each network (**A**) DAN–Dorsal Attention Network, (**B**) CEN–Central Executive Network, (**C**) VAN–Ventral Attention Network, and (**D**) SMN–Sensory Motor Network. The stronger connections in PD compared to HC are in red arrows, the weaker connections in PD compared to HC are in blue arrows.

Within the DAN, PD patients demonstrated lower tPDCs in 9 out of 11 (81 %) significant connections in the theta band; 8 out of 11 (73%) significant connections in the alpha band; and 6 out of 6 (100%) significant connections in the beta band, *p* < 0.046 (Figure 3A, Supplementary Table S3A). Within the CEN, PD patients showed lower tPDCs in 19 out of 22 (86%) significant connections in the theta band; 23 out of 24 (96%) significant connections in the alpha band; and 12 out of 12 (100%) significant connections in the beta band, *p* < 0.01 (Figure 3B, Supplementary Table S3B). In contrast, within the VAN, PD patients demonstrated higher tPDCs in 20 out of 29 (67%) significant connections in the theta band; 35 out of 46 (76 %) significant connections in the alpha band; and 23 out of 38 (61%) significant connections in the beta band, *p* < 0.047 (Figure 3C, Supplementary Table S3C). The tPDCs within the SMN demonstrated mixed patterns between HC and PD. In the theta band, 9 out of 17 (53 %) tPDCs were higher in the PD. In the alpha band, 10 out of 25 (40 %) tPDCs were found to be higher in PD. In the beta band, 9 out of 17 (53 %) tPDCs were higher in PD patients, *p* < 0.048 (Figure 3D, Supplementary Table S3D). No differences in the number of outbound and inbound tPDCs for each node within each network were found between the groups (*p* value DAN > 0.26, *p* value CEN > 0.37, *p* value VAN and SMN > 0.09).

## 4. Discussion

Harnessing the high temporal resolution of EEG, we explored the DFC patterns in major brain networks in early-stage PD. To our knowledge, this is the first study to examine DFC based on EEG resting state networks (RSNs) in PD patients. This unique combination allowed us to investigate directed functional connectivity at a larger range of frequencies, as compared to the very low frequency accessible in fMRI. The obtained results revealed lower DFC in the CEN and DAN in patients with PD compared to HC in all investigated frequency bands. The lower connectivity was detected at two levels; one at the network level demonstrating lower percent of significant tPDCs within the CEN and DAN, and the second at the level of a connection between network’s hub-pairs, showing lower strength in most of the tPDCs. In contrast, in the VAN and SMN we did not detect any differences at the network level–percent of significant tPDCs–between patients with PD and controls. However, we found differences at the level of a connection between network’s hub-pairs. In the VAN, we observed greater strength in most of the tPDCs in PD patients compared to controls. In the SMN, mixed findings were observed, in which, each group demonstrated greater strength in different tPDCs. These findings extend previous work by evaluating connectivity changes in four main cognitive and motor networks in patients with PD using a novel EEG analysis approach.

The DAN and VAN are distinct attentional brain networks implicated in voluntary deployment of attention [40]. The DAN mediates top-down guided voluntary allocation of attention to locations or objects, whereas the VAN is involved in detecting unexpected, but behaviorally relevant stimuli and eliciting shifts of attention [40]. Our obtained EEG findings revealed lower percent of significant connections and reduced strength of each connection in the DAN in patients with PD compared to HCs. This is in line with a previous graph-theory rs-fMRI study, which showed decreased DAN global efficiency and no alterations in VAN connectivity measures even in patients at later stages of the disease [13]. Our findings further extend this knowledge by providing information at the level of a connection between two network’s hub-pairs, revealing weaker strength of connections in the DAN and greater strength of connections in the VAN in patients with PD compared to HCs (recall Figure 3). These findings may explain the impairments in top-down guided voluntary allocation of attention mediated by the DAN, and the intact ability to trigger shift of attention by presenting unexpected external stimuli mediated by the VAN [40]. Interactions between the VAN and DAN allow the dynamic control of attention in relation to top-down goals and bottom-up sensory stimulation [40,41]. Therefore, it is likely that the observed higher DFC connections in the VAN in PD patients serves as a compensatory mechanism to overcome DAN deficits.

Compared to HCs, reduced connectivity in the CEN at the network level and at the connection level in PD patients were observed, further corroborating previous rs-fMRI studies that showed reduced global connectivity within the CEN in patients with PD compared to HCs [42,43]. The CEN plays an important role in working memory, integration of sensory and memory information, and regulation of cognition and behavior [44,45]. Despite these observed changes in CEN connectivity, no indication of reduced cognitive function based on the administered clinical assessments (MOCA and CTT; Table 1) were observed. Therefore, one possible explanation is the lack of sensitivity in our used cognitive tests to detect impairments in executive functions. Alternatively, the greater connectivity observed in the VAN might compensate for the CEN reduced connectivity, reflecting functional flexibility of these networks; i.e., once one network is impaired the other comes into play.

The SMN integrates primary sensorimotor, premotor and supplementary motor areas (SMA) to facilitate voluntary movements [46,47]. fMRI studies demonstrated disease-related changes in this network in PD. However, the reported results vary across studies mainly due to different selected patient samples [16,48,49]. Our current findings provide a possible explanation to the discrepancies between the studies, showing on the one hand a similar connectivity at the network level between PD patients and HCs, but on the other hand significant differences in the strength of specific connections between these groups. Additionally, since no differences between the groups were observed in the number of outbound and inbound tPDCs to any of the network’s hubs, it might be indicative of unchanged information flow at the network level in the early stages of PD. The mixed patterns observed in the SMN might be reflective of the complex dynamic of compensatory mechanisms taking place in the early stages of PD, at which the neural reserve capacities of these patients are relatively preserved.

Finally, only minor differences were observed at the level of the frequency bands within each investigated network. In most cases, these changes in connectivity were at the whole network level, and were found to be unrelated to specific bands. However, as depicted in Figure 3, in the CEN and the DAN there were few connections that were found to be stronger in the PD group in theta and alpha bands. These findings might reflect the increased activity of lower frequencies in PD patients.

The present work has several limitations. The MRI-based templates that were used are based on the population of healthy subjects, therefore, their spatial distribution may not fully resemble this of the studied sample. We used the AAL atlas for parcellation, as it is the most commonly used atlas in MRI studies. However, AAL may lead to changes in parcel homogeneity for functional connectivity. Future studies should examine alternative atlas, such as the Schaefer/Yeo atlas, in which each node is pre-assigned to a functional system based on a cross-validated study. Another limitation is that the PD patients were examined during the ON state, and therefore possible dopaminergic effects on the results cannot be ruled out. Future studies should examine naïve patients or patients during the OFF state to better understand the dopaminergic effects. It is also important to note that our approach was hypothesis-driven. Future studies should also include data-driven approaches and examine the differences between hypothesis-driven and data-driven approaches. Our results encourage a multimodal line of research that combines MRI and EEG to elucidate large-scale cortical dynamic functional network connectivity in PD.

In conclusion, we demonstrate the feasibility to investigate resting-state FC in brain networks using EEG in PD. The obtained findings demonstrate a complex pattern of FC alteration within major brain networks, reflecting the co-occurrence of impairment and compensatory mechanisms processes taking place in the early stages of PD.

## Figures and Tables

**Table 1:** Participants’ Characteristics.

	HC (n = 28)	PD (n = 29)	*p* Values
Age (years)	60.82 ± 7.47	64.36 ± 7.11	0.075
Gender (male/female)	13/15	16/13	0.518
MoCA	27.44 ± 1.97	26.77 ± 2.1	0.104
CTT (part B)	84.85 ± 29.66	101.56 ± 57.03	0.186
MDS-UPDRS motor	1.38 ± 1.74	18.89 ± 10.05	**<0.001**
Disease duration (years)	na	2.8 ± 0.28	na
LEDD (mg)	na	183.90 ± 249.0	na
HY score (HY1/HY2)	na	10/19	na

HC = Healthy controls, PD = Parkinson’s disease, MOCA = Montreal cognitive assessment, CTT = Color trial test, MDS-UPDRS = Unified Parkinson’s disease rating scale, LEDD = Levodopa equivalent daily doses.

## Data Availability

Data and code will be available upon request from the authors. The codes for producing the tPDC are available in the following link: https://github.com/yaelezra/tPDC.git. accessed on: 8 February 2022.

## Supplementary Materials

The following supporting information can be downloaded at: https://www.mdpi.com/article/10.3390/biomimetics7040231/s1, Table S1 A/B/C/D: Definition of network hubs in the CEN/DAN/VAN/SMN using anatomy and Brodmann Areas; Table S2: Post hoc analysis for the differences in percent of significant tPDC between the four RSNs; Table S3 A/B/C/D: Averaged tPDC values for each group in the DAN/CEN/VAN/SMN (PD > HC in red, HC > PD in blue).
