# Supplementary material for: EEG-Based Mapping of Resting-State Functional Brain Networks in Patients with Parkinson’s Disease
Source: Biomimetics (Basel). 2022 Dec 8;7(4):231. doi: 10.3390/biomimetics7040231 (PMC9775055; doi:10.3390/biomimetics7040231)
Supplement: Supplementary file 1 [file biomimetics-07-00231-s001.zip › biomimetics-2061606-supplementary.pdf]

Supplementary Materials

Table S1 A: Definition of network hubs in the CEN using anatomy and Brodmann Areas

Brodmann Area	Anatomy	Acronym
6	Supplementary motor area	SMA
7	Superior parietal gyrus	SPG
32 + 8	Prefrontal motor cortex, Pregenual anterior cingulate cortex	Pre-SMA, prACC
46 + 9	Median prefrontal cortex, Dorso lateral prefrontal cortex	MPFC, DLPFC
10	Anterior lateral prefrontal cortex	ALPFC
47 + 11	Ventro-lateral prefrontal cortex, Orbital frontal cortex	VLPFC, OFC
20 + 21 + 37	Inferior and middle temporal gyrus, Occipital-temporal cortex	ITG, MTG, OTC
23 + 24	Posterior cingulate cortex1, Dorsal anterior cingulate cortex	PCC1, DACC
22 + 39 + 40	Superior temporal gyrus, Angular gyrus, Intra-parietal sulcus	STG, AG, IPS
44 + 45	Opercular part of inferior frontal gyrus, Inferior frontal gyrus	OPCG, IFG
48		BA 48

Table S1 B: Definition of network hubs in the DAN using anatomy and Brodmann Areas

Brodmann Area	Anatomy	Acronym
1 + 2 + 3	Primary, secondary and tertiary somatosensory cortex	S1, S2, S3
5	Secondary somatosensory sulcus	SPS
6 + 8	Supplementary motor area, Pre-supplementary motor area	SMA, Pre-SMA
7	Superior parietal gyrus	SPG
18 + 19	Secondary visual cortex, Cuneus	V2, Cuneus
20 + 21 + 37	Inferior and middle temporal gyrus, Occipital-temporal cortex	ITG, MTG, OTC
39 + 40	Angular gyrus, Intra-parietal sulcus	AG, IPS
44	Opercular part of inferior frontal gyrus	OPCG
48		BA48

Table S1 C: Definition of network hubs in the VAN using anatomy and Brodmann Areas

Brodmann Area	Anatomy	Acronym
1 + 2 + 3 + 5	Primary, secondary and tertiary somatosensory cortex, Superior parietal gyrus	S1, S2, S3, SPS
6	Supplementary motor area	SMA
46 + 9	Medial prefrontal cortex, Dorso lateral prefrontal cortex	MPFC, DLPFC
47 + 11	Ventro-lateral prefrontal cortex, Orbital frontal cortex	VLPFC, OFC
20 + 21 + 37	Inferior and middle temporal gyrus, Occipital-temporal cortex	ITG, MTG, OTC
22 + 39 + 40	Superior temporal gyrus, Angular gyrus, Intra-parietal sulcus	STG, AG, IPS
23 + 24	Posterior cingulate cortex1	PCC1, DACC
32	Pregenual anterior cingulate cortex	prACC
41 + 42	Primary and secondary auditory cortex	A1, A2
44 + 45	Opercular part of inferior frontal gyrus, Inferior frontal gyrus	OPCG, IFG
48		BA 48

Table S1 D: Definition of network hubs in the SMN using anatomy and Brodmann Areas

Brodmann Area	Anatomy	Acronym
1 + 2 + 3 + 5	Primary, secondary and tertiary somatosensory cortex, Superior parietal gyrus	S1, S2, S3, SPS
4	Primary motor cortex	M1
6	Supplementary motor area	SMA
21	Middle temporal gyrus	MTG
22 + 40	Superior temporal gyrus, Intra-parietal sulcus	STG, IPS
23 + 24	Posterior cingulate cortex1	PCC1, DACC
38	Temporal pole	TP
41 + 42	Primary and secondary auditory cortex	A1, A2
43	Postcentral gyrus	PCG
48		BA 48

Table S2: Post-Hoc analysis for the differences in percent of significant tPDC between the four RSNs

(I) Network	(J) Network	Mean Difference (I-J)	Std. Error	df	Sig.[c]	95% Confidence Interval for Difference[c]	
						Lower Bound	Upper Bound
CEN	DAN	-.037*	.006	627.000	.000	-.049	-.026
	VAN	-.115*	.006	627.000	.000	-.127	-.104
	SMN	-.094*	.006	627.000	.000	-.106	-.083
DAN	CEN	.037*	.006	627.000	.000	.026	.049
	VAN	-.078*	.006	627.000	.000	-.089	-.066
	SMN	-.057*	.006	627.000	.000	-.068	-.045
VAN	CEN	.115*	.006	627.000	.000	.104	.127
	DAN	.078*	.006	627.000	.000	.066	.089
	SMN	.021*	.006	627.000	.000	.009	.032
SMN	CEN	.094*	.006	627.000	.000	.083	.106
	DAN	.057*	.006	627.000	.000	.045	.068
	VAN	-.021*	.006	627.000	.000	-.032	-.009

*significant at the .05 level, a. Dependent Variable: RSN, c. Adjustment for multiple comparisons.

Table S3 A: Averaged tPDC values for each group in the DAN (PD>HC in red, HC>PD in blue)

		Group	
**band**	**Connection**	HC	PD
**theta**	AG, IPS->SPS	0.165062	0.841119
	BA 48->OPCG	0.003678	0.005037
	SMA, Pre-SMA->SPG	0.081502	0.073379
	SPG->SPS	0.176137	0.158872
	ITG, MTG, OTC->S1, S2, S3	0.008746	0.007248
	ITG, MTG, OTC->SPS	0.111137	0.086914
	ITG, MTG, OTC->SMA, Pre-SMA	0.013424	0.010598
	ITG, MTG, OTC->SPG	0.048775	0.037298
	AG, IPS->SMA, Pre-SMA	0.026156	0.024814
	OPCG->SPG	0.072214	0.065742
	BA 48->SPG	0.084869	0.07343
**alpha**	S1, S2, S3->SPS	0.155918	0.176483
	S1, S2, S3->SMA, Pre-SMA	0.01817	0.019479
	BA 48->SMA, Pre-SMA	0.020515	0.021926
	SMA, Pre-SMA->SPG	0.078535	0.073639
	V2, Cuneus->S1, S2, S3	0.011762	0.011148
	V2, Cuneus->SPS	0.177187	0.170688
	ITG, MTG, OTC->SMA, Pre-SMA	0.012309	0.010048
	ITG, MTG, OTC->SPG	0.047321	0.036748
	AG, IPS->SPG	0.02247	0.021123
	OPCG->SPG	0.072582	0.065844
	BA 48->SPG	0.075564	0.070626
**beta**	SMA, Pre-SMA->SPS	0.155838	0.152654
	ITG, MTG, OTC->SMA, Pre-SMA	0.011792	0.008734
	AG, IPS->SPG	0.077027	0.068891
	OPCG->SPS	0.178938	0.169326
	OPCG->SPG	0.076796	0.069918
	BA 48->SPS	0.16947	0.158813

Table S3 B: Averaged tPDC values for each group in the CEN (PD>HC in red, HC>PD in blue)

		Group	
**band**	Connection	HC	PD
**theta**	ALPFC->VLPFC, OFC	0.027856	0.030985
	PCC1, DACC->Pre-SMA, prACC	0.014274	0.015479
	BA 48->VLPFC, OFC	0.035524	0.04075
	SMA->Pre-SMA, prACC	0.016614	0.01281
	SMA->ALPFC	0.003781	0.002969
	SMA->VLPFC, OFC	0.032847	0.031222
	SMA-> STG, AG, IPS	0.014524	0.010905
	SPG->SMA	0.010936	0.008078
	SPG->STG, AG, IPS	0.012446	0.011548
	MPFC, DLPFC->SMA	0.010112	0.009144
	ALPFC->MPFC, DLPFC	0.007965	0.007002
	VLPFC, OFC->SMA	0.009032	0.006302
	VLPFC, OFC->SPG	0.022775	0.017628
	VLPFC, OFC->Pre-SMA, prACC	0.012861	0.009675
	VLPFC, OFC->ALPFC	0.005412	0.003669
	VLPFC, OFC->STG, AG, IPS	0.012633	0.009014
	ITG, MTG, OTC->VLPFC, OFC	0.024838	0.018922
	PCC1, DACC->SMA	0.01371	0.012098
	STG, AG, IPS ->Pre-SMA, prACC	0.014021	0.012082
	STG, AG, IPS ->ALPFC	0.003117	0.002882
	STG, AG, IPS ->VLPFC, OFC	0.033338	0.0267
	BA 48->STG, AG, IPS	0.012401	0.011434
**alpha**	OPCG, IFG->MPFC, DLPFC	0.012729	0.013786
	SMA->ALPFC	0.003478	0.002763
	SMA->STG, AG, IPS	0.014939	0.009784
	SPG->Pre-SMA, prACC	0.014784	0.01101
	Pre-SMA, prACC->ALPFC	0.003125	0.002304
	MPFC, DLPFC->SMA	0.003929	0.003624
	MPFC, DLPFC->VLPFC, OFC	0.010317	0.008888
	MPFC, DLPFC->STG, AG, IPS	0.031031	0.027145
	ALPFC->SMA	0.013517	0.009939
	ALPFC->VLPFC, OFC	0.010564	0.007896
	VLPFC, OFC->SPG	0.008473	0.006937
	VLPFC, OFC->Pre-SMA, prACC	0.022638	0.018974
	ITG, MTG, OTC->Pre-SMA, prACC	0.011917	0.009865

	ITG, MTG, OTC->VLPFC, OFC	0.006807	0.005398
	PCC1, DACC->SPG	0.020544	0.016301
	STG, AG, IPS ->SMA	0.027822	0.020953
	STG, AG, IPS ->SPG	0.0093	0.008468
	STG, AG, IPS ->Pre-SMA, prACC	0.025067	0.022637
	STG, AG, IPS ->ALPFC	0.012113	0.011361
	OPCG, IFG->SMA	0.003119	0.002927
	OPCG, IFG->SPG	0.011297	0.010809
	OPCG, IFG->ALPFC	0.012816	0.01157
	OPCG, IFG-> STG, AG, IPS	0.004081	0.003834
	BA 48->Pre-SMA, prACC	0.012697	0.011182
**beta**	SMA-> STG, AG, IPS	0.013386	0.009655
	SPG->Pre-SMA, prACC	0.012582	0.011122
	Pre-SMA, prACC->SPG	0.02534	0.022516
	Pre-SMA, prACC->ALPFC	0.003679	0.003173
	MPFC, DLPFC->STG, AG, IPS	0.012446	0.010562
	ITG, MTG, OTC->SMA	0.005636	0.004084
	ITG, MTG, OTC->VLPFC, OFC	0.022158	0.018074
	PCC1, DACC->SPG	0.0331	0.024536
	PCC1, DACC->Pre-SMA, prACC	0.016392	0.013535
	STG, AG, IPS ->SPG	0.027397	0.021954
	OPCG, IFG->ALPFC	0.00417	0.003863
	OPCG, IFG-> STG, AG, IPS	0.012495	0.010613

Table S3 C: Averaged tPDC values for each group in the VAN (PD>HC in red, HC>PD in blue)

		Group	
**band**	Connection	HC	PD
**theta**	S1, S2, S3, SPS->VLPFC, OFC	0.038812	0.045667
	SMA->S1, S2, S3, SPS	0.074118	0.076505
	SMA->STG, AG, IPS	0.117284	0.141681
	MPFC, DLPFC->ITG, MTG, OTC	0.075305	0.085393
	MPFC, DLPFC->STG, AG, IPS	0.11335	0.129263
	MPFC, DLPFC->BA 48	0.014331	0.017096
	VLPFC, OFC->STG, AG, IPS	0.12408	0.143758
	VLPFC, OFC->A1, A2	0.025426	0.028204
	ITG, MTG, OTC->A1, A2	0.023329	0.025081
	STG, AG, IPS->SMA	0.141363	0.155686
	STG, AG, IPS->ITG, MTG, OTC	0.091686	0.102521
	STG, AG, IPS->PCC1, DACC	0.011053	0.012651
	STG, AG, IPS->A1, A2	0.024315	0.026818
	STG, AG, IPS->BA 48	0.022324	0.02557
	PCC1, DACC->BA 48	0.018093	0.018961
	prACC->S1, S2, S3, SPS	0.079102	0.086482
	prACC->OPCG, IFG	0.057901	0.064902
	A1, A2->BA 48	0.016888	0.021131
	OPCG, IFG->S1, S2, S3, SPS	0.09215	0.096635
	OPCG, IFG->A1, A2	0.021438	0.02693
	S1, S2, S3, SPS->MPFC, DLPFC	0.197328	0.16397
	MPFC, DLPFC->SMA	0.139404	0.123676
	MPFC, DLPFC->VLPFC, OFC	0.044181	0.038422
	VLPFC, OFC->SMA	0.165549	0.147758
	ITG, MTG, OTC->MPFC, DLPFC	0.213123	0.175946
	STG, AG, IPS->S1, S2, S3, SPS	0.09539	0.081995
	PCC1, DACC->prACC	0.033422	0.030445
	prACC->MPFC, DLPFC	0.207296	0.191777
	A1, A2->SMA	0.154582	0.140783
**alpha**	S1, S2, S3, SPS->VLPFC, OFC	0.042966	0.04687
	S1, S2, S3, SPS->OPCG, IFG	0.057482	0.064588
	SMA->ITG, MTG, OTC	0.085385	0.097471
	SMA->STG, AG, IPS	0.119706	0.143383
	SMA->A1, A2	0.02418	0.026441
	SMA->OPCG, IFG	0.044313	0.051071

	SMA->BA 48	0.020821	0.0221
	MPFC, DLPFC->ITG, MTG, OTC	0.079197	0.088296
	MPFC, DLPFC->STG, AG, IPS	0.113635	0.128475
	MPFC, DLPFC->prACC	0.022263	0.023397
	MPFC, DLPFC->BA 48	0.015172	0.018154
	VLPFC, OFC->STG, AG, IPS	0.121081	0.140821
	VLPFC, OFC->A1, A2	0.024255	0.026791
	ITG, MTG, OTC->STG, AG, IPS	0.113494	0.137682
	ITG, MTG, OTC->A1, A2	0.023282	0.025237
	STG, AG, IPS->SMA	0.147693	0.154757
	STG, AG, IPS->ITG, MTG, OTC	0.09515	0.104476
	STG, AG, IPS->PCC1, DACC	0.014835	0.015997
	STG, AG, IPS->A1, A2	0.024541	0.028306
	STG, AG, IPS->OPCG, IFG	0.075045	0.079609
	PCC1, DACC->STG, AG, IPS	0.143965	0.15967
	prACC->S1, S2, S3, SPS	0.077081	0.080872
	prACC->A1, A2	0.032626	0.037358
	prACC->OPCG, IFG	0.058315	0.068053
	A1, A2->S1, S2, S3, SPS	0.08788	0.091456
	A1, A2->STG, AG, IPS	0.129336	0.141477
	A1, A2->BA 48	0.016866	0.022059
	OPCG, IFG->S1, S2, S3, SPS	0.084277	0.088126
	OPCG, IFG->ITG, MTG, OTC	0.08954	0.098357
	OPCG, IFG->STG, AG, IPS	0.131995	0.145958
	OPCG, IFG->BA 48	0.018464	0.020665
	BA 48->ITG, MTG, OTC	0.094532	0.102405
	BA 48->STG, AG, IPS	0.161338	0.165834
	BA 48->prACC	0.032994	0.034273
	BA 48->A1, A2	0.024615	0.025568
	S1, S2, S3, SPS->SMA	0.156634	0.147743
	S1, S2, S3, SPS->MPFC, DLPFC	0.209815	0.16552
	SMA->prACC	0.029612	0.028016
	MPFC, DLPFC->S1, S2, S3, SPS	0.072	0.063245
	MPFC, DLPFC->SMA	0.132357	0.126287
	MPFC, DLPFC->VLPFC, OFC	0.041938	0.039489
	ITG, MTG, OTC->SMA	0.160842	0.150335
	ITG, MTG, OTC->MPFC, DLPFC	0.225312	0.190294
	STG, AG, IPS->MPFC, DLPFC	0.202571	0.178051
	OPCG, IFG->SMA	0.154295	0.139485
	BA 48->MPFC, DLPFC	0.198181	0.183529
**beta**	S1, S2, S3, SPS->VLPFC, OFC	0.03925	0.041495

S1, S2, S3, SPS->STG, AG, IPS	0.129371	0.135208
SMA->STG, AG, IPS	0.122397	0.134566
SMA->OPCG, IFG	0.051191	0.055433
MPFC, DLPFC->STG, AG, IPS	0.114776	0.123831
VLPFC, OFC->ITG, MTG, OTC	0.102351	0.104463
VLPFC, OFC->prACC	0.028587	0.029317
VLPFC, OFC->A1, A2	0.024496	0.025412
ITG, MTG, OTC->S1, S2, S3, SPS	0.088135	0.092943
ITG, MTG, OTC->A1, A2	0.027597	0.030245
STG, AG, IPS->ITG, MTG, OTC	0.094601	0.10189
PCC1, DACC->ITG, MTG, OTC	0.098846	0.104746
PCC1, DACC->STG, AG, IPS	0.140701	0.14855
PCC1, DACC->prACC	0.034722	0.03677
PCC1, DACC->BA 48	0.020018	0.020653
prACC->ITG, MTG, OTC	0.099225	0.105332
prACC->STG, AG, IPS	0.132566	0.143385
prACC->A1, A2	0.029901	0.032287
A1, A2->ITG, MTG, OTC	0.089501	0.102951
A1, A2->OPCG, IFG	0.053638	0.067312
A1, A2->BA 48	0.020504	0.022652
OPCG, IFG->STG, AG, IPS	0.133818	0.146892
BA 48->STG, AG, IPS	0.147929	0.161936
S1, S2, S3, SPS->MPFC, DLPFC	0.202251	0.167243
SMA->S1, S2, S3, SPS	0.076856	0.072881
SMA->MPFC, DLPFC	0.204527	0.180016
SMA->BA 48	0.019973	0.019254
MPFC, DLPFC->SMA	0.128873	0.118377
VLPFC, OFC->OPCG, IFG	0.060534	0.058969
ITG, MTG, OTC->MPFC, DLPFC	0.229944	0.202678
ITG, MTG, OTC->VLPFC, OFC	0.046114	0.040381
STG, AG, IPS->MPFC, DLPFC	0.205045	0.182689
PCC1, DACC->MPFC, DLPFC	0.190446	0.179004
PCC1, DACC->VLPFC, OFC	0.046869	0.044612
prACC->SMA	0.161853	0.139922
A1, A2->VLPFC, OFC	0.057846	0.054627
A1, A2->PCC1, DACC	0.012768	0.011779
OPCG, IFG->S1, S2, S3, SPS	0.101987	0.094494

Table S3 D: Averaged tPDC values for each group in the SMN (PD>HC in red, HC>PD in blue)

		Group	
**band**	Connection	HC	PD
**theta**	S1, S2, S3, SPS->M1	0.047951	0.054828
	S1, S2, S3, SPS->SMA	0.089281	0.094602
	M1->TP	0.126988	0.131198
	SMA->TP	0.128104	0.139203
	MTG->PCG	0.015475	0.019724
	PCC1, DACC->PCG	0.016522	0.017628
	A1, A2->PCG	0.014492	0.016817
	PCG->TP	0.13035	0.139825
	BA 48->TP	0.128592	0.142052
	S1, S2, S3, SPS->PCC1, DACC	0.023812	0.020183
	MTG->M1	0.051833	0.045091
	MTG->PCC1, DACC	0.02583	0.018465
	STG, IPS->PCC1, DACC	0.02625	0.022778
	PCC1, DACC->SMA	0.087935	0.075914
	PCC1, DACC->A1, A2	0.015043	0.014521
	A1, A2->SMA	0.07845	0.074159
	PCG->S1, S2, S3, SPS	0.11153	0.103372
**alpha**	S1, S2, S3, SPS->TP	0.129829	0.13478
	SMA->TP	0.121678	0.142537
	MTG->PCG	0.016287	0.021375
	PCC1, DACC->PCG	0.013569	0.014815
	TP->SMA	0.075711	0.078069
	TP->MTG	0.326448	0.339547
	TP->A1, A2	0.014164	0.014845
	A1, A2->PCG	0.017544	0.01839
	PCG->SMA	0.073949	0.078183
	BA 48->TP	0.124223	0.13653
	S1, S2, S3, SPS->STG, IPS	0.087774	0.082281
	M1->TP	0.135821	0.131458
	SMA->M1	0.049551	0.044226
	MTG->S1, S2, S3, SPS	0.100692	0.08819
	MTG->M1	0.052388	0.044139
	MTG->SMA	0.076138	0.070265
	MTG->PCC1, DACC	0.024125	0.018915
	STG, IPS->S1, S2, S3, SPS	0.105324	0.096035

	PCC1, DACC->SMA	0.096486	0.0749
	PCC1, DACC->A1, A2	0.014146	0.012747
	A1, A2->S1, S2, S3, SPS	0.109753	0.096255
	A1, A2->SMA	0.081597	0.074963
	A1, A2->PCC1, DACC	0.025052	0.023942
	PCG->M1	0.056777	0.05069
	BA 48->PCC1, DACC	0.026524	0.020676
	BA 48->PCG	0.016252	0.015401
**beta**	S1, S2, S3, SPS->M1	0.04695	0.048393
	S1, S2, S3, SPS->PCG	0.016456	0.017378
	MTG->TP	0.133657	0.138219
	MTG->PCG	0.017038	0.018233
	STG, IPS->TP	0.134899	0.139487
	PCC1, DACC->PCG	0.015714	0.016599
	TP->SMA	0.068748	0.072836
	A1, A2->STG, IPS	0.094761	0.098122
	BA 48->TP	0.141094	0.147729
	SMA->S1, S2, S3, SPS	0.103607	0.093513
	MTG->PCC1, DACC	0.025301	0.021045
	TP->M1	0.050249	0.048176
	A1, A2->M1	0.04911	0.047285
	A1, A2->SMA	0.091527	0.081437
	A1, A2->PCC1, DACC	0.03626	0.02933
	PCG->SMA	0.073397	0.070638
	BA 48->PCC1, DACC	0.025998	0.019467
